# Nasal gene expression differentiates COPD from controls and overlaps bronchial gene expression

**DOI:** 10.1186/s12931-017-0696-5

**Published:** 2017-12-21

**Authors:** Ilse M. Boudewijn, Alen Faiz, Katrina Steiling, Erica van der Wiel, Eef D. Telenga, Susan J. M. Hoonhorst, Nick H. T. ten Hacken, Corry-Anke Brandsma, Huib A. M. Kerstjens, Wim Timens, Irene H. Heijink, Marnix R. Jonker, Harold G. de Bruin, J. Sebastiaan Vroegop, Henk R. Pasma, Wim G. Boersma, Pascal Wielders, Frank van den Elshout, Khaled Mansour, Avrum Spira, Marc E. Lenburg, Victor Guryev, Dirkje S. Postma, Maarten van den Berge

**Affiliations:** 1 University of Groningen, University Medical Center Groningen, Department of Pulmonary Diseases, Groningen, the Netherlands; 2 University of Groningen, University Medical Center Groningen, Groningen Research Institute for Asthma and COPD, Groningen, the Netherlands; 3 Division of Computational Biomedicine, Boston University School of Medicine, Boston, MA USA; 4 Bioinformatics Program, Boston University, Boston, MA USA; 5 University of Groningen, University Medical Center Groningen, Department of Pathology, section Medical Biology, Groningen, the Netherlands; 6 Martini Hospital, Department of Pulmonary Diseases, Groningen, the Netherlands; 7 Medical Center Leeuwarden, Department of Pulmonary Diseases, Leeuwarden, the Netherlands; 8 Noordwest Ziekenhuisgroep, Department of Pulmonary Diseases, Alkmaar, the Netherlands; 9 Catharina Hospital, Department of Pulmonary Diseases, Eindhoven, the Netherlands; 10 Rijnstate Hospital, Department of Pulmonary Diseases, Arnhem, the Netherlands; 11 Orbis Concern, Department of Pulmonary Diseases, Sittard, the Netherlands; 12 European Research Institute for the Biology of Ageing, University of Groningen, University Medical Center Groningen, Groningen, the Netherlands

**Keywords:** COPD, Nasal epithelium, Bronchial epithelium, Genome wide gene expression, Microarray

## Abstract

**Background:**

Nasal gene expression profiling is a promising method to characterize COPD non-invasively. We aimed to identify a nasal gene expression profile to distinguish COPD patients from healthy controls. We investigated whether this COPD-associated gene expression profile in nasal epithelium is comparable with the profile observed in bronchial epithelium.

**Methods:**

Genome wide gene expression analysis was performed on nasal epithelial brushes of 31 severe COPD patients and 22 controls, all current smokers, using Affymetrix Human Gene 1.0 ST Arrays. We repeated the gene expression analysis on bronchial epithelial brushes in 2 independent cohorts of mild-to-moderate COPD patients and controls.

**Results:**

In nasal epithelium, 135 genes were significantly differentially expressed between severe COPD patients and controls, 21 being up- and 114 downregulated in COPD (false discovery rate < 0.01). Gene Set Enrichment Analysis (GSEA) showed significant concordant enrichment of COPD-associated nasal and bronchial gene expression in both independent cohorts (FDR_GSEA_ < 0.001).

**Conclusion:**

We identified a nasal gene expression profile that differentiates severe COPD patients from controls. Of interest, part of the nasal gene expression changes in COPD mimics differentially expressed genes in the bronchus. These findings indicate that nasal gene expression profiling is potentially useful as a non-invasive biomarker in COPD.

**Trial registration:**

ClinicalTrials.gov registration number NCT01351792 (registration date May 10, 2011), ClinicalTrials.gov registration number NCT00848406 (registration date February 19, 2009), ClinicalTrials.gov registration number NCT00807469 (registration date December 11, 2008).

**Electronic supplementary material:**

The online version of this article (10.1186/s12931-017-0696-5) contains supplementary material, which is available to authorized users.

## Background

Chronic obstructive pulmonary disease (COPD) is one of the most common chronic diseases affecting over 10% of adults older than 65 years worldwide [1]. It is characterized by incompletely reversible airflow obstruction, which results in reduced quality of life and increased mortality. COPD is currently the fourth leading cause of death worldwide, according to the World Health Organization [2].

Some genetic factors, such as alpha-1 anti-trypsin deficiency, and environmental exposures, such as cigarette smoking, are well-established contributors to COPD, yet the precise molecular mechanisms of the disease remain to be elucidated. Genome-wide gene expression profiling offers opportunities to develop new insights in the origins of COPD based on molecular features.

The airway epithelium is the initial site of exposure to cigarette smoke and reflects molecular changes associated with both cigarette smoke exposure and smoking-associated lung disease. We have previously demonstrated that the bronchial airway epithelium responds to cigarette smoke exposure with characteristic alterations in gene expression [3]. Building upon this observation, we identified a bronchial airway gene expression signature associated with COPD and disease severity that is similarly altered in COPD-affected lung tissue [4]. These data suggest that bronchial gene expression obtained by bronchoscopy may be applied to monitor disease activity in COPD. However, the relative invasiveness of this procedure precludes its use in large populations. We therefore characterized the upper airway genomic response to cigarette smoke exposure by profiling epithelial cells collected by brushing the inferior turbinate of the nose. Using paired bronchial and nasal airway samples obtained from the same healthy individuals, we showed that smoking-induced changes in bronchial airway gene expression are similarly altered in the nasal epithelium [5]. Therefore, nasal epithelial gene expression profiling could potentially function as a non-invasive biomarker in COPD.

In the present study, we investigated whether gene expression profiling of the nasal epithelium can distinguish between patients with and without COPD. Additionally, we established whether COPD-associated gene expression changes in nasal epithelium reflect those occurring in the bronchial epithelium.

## Methods

The online supplement provides additional details and a flowchart of the identification cohort and comparator cohorts (online Additional file 1: Figure E1).

### Identification cohort, sample collection and processing

The local ethical committees approved the studies and all included subjects gave their written informed consent. COPD patients were recruited in the University Medical Center Groningen (UMCG) and seven other participating hospitals in The Netherlands between 2011 and 2012. From this study, we only included current smokers aged 40 years and older with at least 10 pack years, a forced expiratory volume in 1 s (FEV_1_)/forced vital capacity (FVC) < 0.7 and an FEV_1_ < 60% predicted. Controls were recruited in the UMCG between 2009 and 2012, including only current smokers aged 40 years and older with normal pulmonary function (i.e. post-bronchodilator FEV_1_/FVC > lower limit of normal, absence of bronchial hyperresponsiveness and reversibility of FEV_1_ to salbutamol <10% predicted). Spirometry and body plethysmography measurements were performed according to international guidelines [6, 7]. Symptom scores were assessed using the Clinical COPD Questionnaire (CCQ) [8].

Nasal epithelial brushings were obtained using a soft cytology brush sampling the inferior turbinate of the nose [5, 9]. Total RNA was isolated and microarray hybridization to Affymetrix Human Gene 1.0 ST Arrays was performed.

### Data normalization, preprocessing and analysis

Statistical analyses were performed using R version 3.3.2. The quality of the microarray hybridization was assessed as previously described [4]. Nasal gene expression profiles associated with COPD were identified using a linear regression model with the log2-transformed expression of each gene as dependent variable, and COPD, age, gender, packyears, RNA integrity number (RIN), and the first 4 principal components (PCs) as independent variables. We adjusted for PCs to reduce technical variation in the microarray data (online Additional file 1). A Benjamini-Hochberg False Discovery Rate (FDR) procedure was applied to account for multiple testing, with an FDR < 0.01 indicating statistical significance [10].

### Comparison of COPD-associated gene expression changes in the nasal and bronchial epithelium

We compared nasal COPD-associated gene expression with bronchial COPD-associated gene expression in two independent datasets of bronchial brushings (comparator cohort 1 and comparator cohort 2 as described below), using Gene Set Enrichment Analysis (GSEA) version 2.2.4 [11]. The local ethical committees approved the two studies and all included subjects gave their written informed consent.

- Comparator cohort 1 was a previously published dataset of bronchial airway gene expression in current and former smokers with and without moderate-to-severe COPD (GSE37147) [4].
- Comparator cohort 2 was an independent cohort of current and former smokers with and without moderate-to-severe COPD who participated in a previous study in the UMCG [12, 13]. COPD was defined as FEV_1_/FVC ≤ 0.7. Bronchial brushes were taken during bronchoscopy and RNA was isolated and processed as described in the online supplement.

We performed GSEA for genes associated with COPD in nasal and bronchial epithelium. First, we investigated COPD-associated nasal gene expression with COPD-associated bronchial gene expression. To this end, bronchial genes were ranked according to the strength of their association with COPD (t-value), and compared to significantly COPD-associated up- and downregulated genes in nasal epithelium.

### Pathway analysis

We compared COPD-associated nasal and bronchial gene expression to gene-sets generated from the Kyoto Encyclopedia of Genes and Genomes (KEGG). A FDR_GSEA_ < 0.25 was considered statistically significant. In addition, to identify more specific biological processes we performed Gene Ontology enrichment analyses (GOrilla) on COPD-associated genes exhibiting differential expression in nasal samples.

## Results

### Study populations

The identification cohort consisted of 31 COPD patients and 22 controls with nasal epithelial brushes. The comparator cohorts contained in total 97 COPD patients and 171 controls with bronchial epithelial brushes. Clinical characteristics are presented in Table 1.

**Table 1:** Characteristics of the identification cohort (nasal epithelial brushes) and comparator cohorts (bronchial epithelial brushes)

	Identification cohort					Comparator cohorts									
	Nasal brushes					Bronchial brushes (cohort 1)					Bronchial brushes (cohort 2)				
	COPD ( *n* = 31)		Control (*n* = 22)		*p*	COPD ( *n* = 87)		Control ( *n* = 151)		*p*	COPD ( *n* = 10)		Control (*n* = 20)		*p*
Age, years	61	(8)	52	(8)	<0.01	65	(6)	64	(6)	0.25	67	(5)	55	(10)	<0.01
Male gender, *n* (%)	21	(68%)	14	(64%)	0.76	52	(60%)	83	(55%)	0.50	10	(100%)	17	(85%)	0.20
Smoking status															
Current smoking, *n* (%)	31	(100%)	22	(100%)	1	30	(35%)	69	(46%)	0.10	8	(80%)	19	(95%)	0.20
Ex smoking, *n* (%)	0	(0%)	0	(0%)		57	(66%)	82	(54%)		2	(20%)	1	(5%)	
Packyears	39	(18)	29	(12)	0.03	51 ^a^	(25)	47 ^a^	(19)	0.11	31 ^b^	(22-45)	26 ^b^	(24-36)	0.57
ICS use, *n* (%)	27	(87%)	–		<0.01	18	(21%)	7	(5%)	<0.01	8	(80%)	0	(0%)	<0.01
FEV _1_^c^ , % predicted	31	(6)	102	(11)	<0.01	60	(14)	93	(13)	<0.01	62 ^b^	(52-68)	102 ^b^	(97-114)	<0.01
FEV _1_ /FVC, %	34	(8)	75	(4)	<0.01	56	(9)	75	(6)	<0.01	50 ^b^	(42-56)	73 ^b^	(71-83)	<0.01
GOLD classification ^c^															
GOLD 1, *n* (%)	0	(0%)	–		–	0	(0%)	–		–	1	(10%)	–		–
GOLD 2, *n* (%)	2	(6%)	–		–	68	(78%)	–		–	8	(80%)	–		–
GOLD 3, *n* (%)	22	(71%)	–		–	17	(20%)	–		–	1	(10%)	–		–
GOLD 4, *n* (%)	7	(23%)	–		–	2	(2%)	–		–	0	(0%)	–		–
TLC, % predicted	132	(17)	105	(10)	<0.01	n/a		n/a		–	n/a		n/a		–
RV/TLC, %	62	(7)	30	(3)	<0.01	n/a		n/a		–	n/a		n/a		–
CCQ	2.4	(0.9)	0.4	(0.3)	<0.01	n/a		n/a		–	n/a		n/a		–
Allergic rhinitis, *n* (%)	1	(3%)	5	(23%)	0.07	n/a		n/a		–	n/a		n/a		–
Nasal CS use, *n* (%)	2	(7%)	1	(5%)	0.99	n/a		n/a		–	n/a		n/a		–

All values are presented as mean with standard deviation, unless stated otherwise; *p* -values reflect differences between COPD and controls

^a^ Missing packyears for 5 subjects with COPD and 11 controls

^b^ Median with interquartile range

^c^ postbronchodilator, except for 1 subject in the identification cohort for which postbronchodilator FEV _1_ was not available, we show prebronchodilator FEV _1_ instead (32%); for all subjects in cohort 1, only prebronchodilator FEV _1_ was available

*ICS* Inhaled corticosteroids, *FEV*_*1*_ Forced expiratory volume in 1 s, *FVC* Forced vital capacity, *GOLD* Global initiative for chronic obstructive lung disease, *TLC* Total lung capacity, *RV* Residual volume, *CCQ* Clinical COPD Questionnaire, *CS* Corticosteroids, *n/a* Not available

### COPD-associated genes in nasal epithelium

**Fig. 1:**
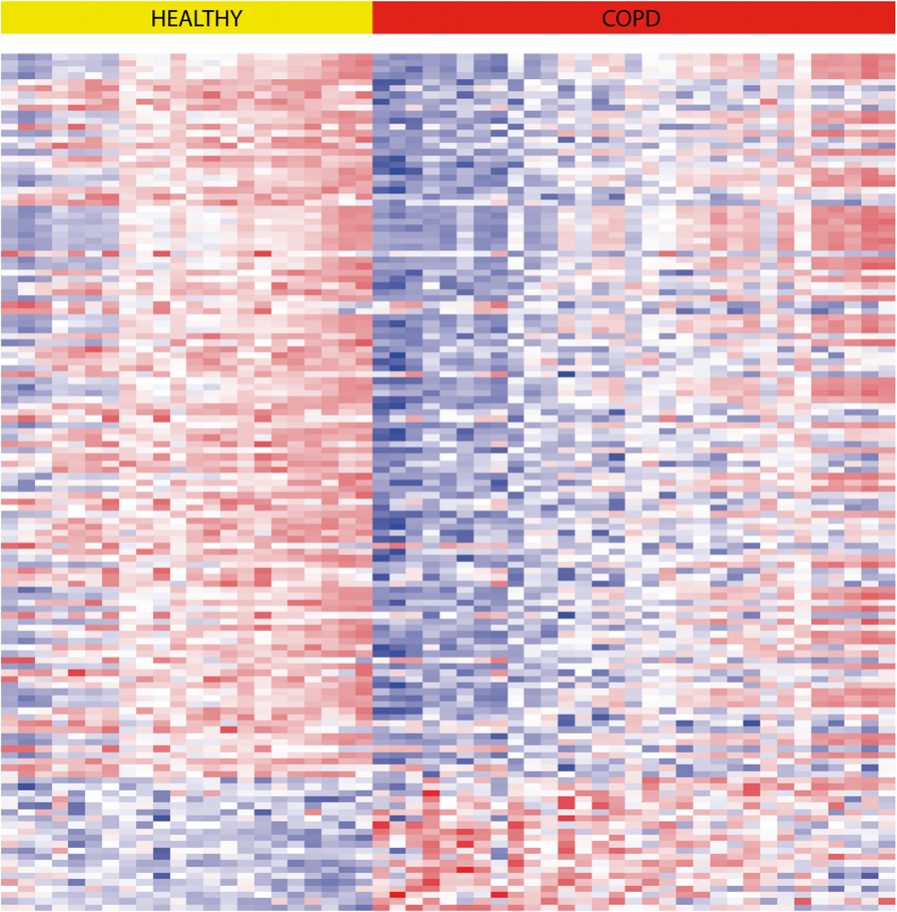
Heatmap of gene expression significantly associated with COPD status. Between COPD and controls, 135 genes were significantly differentially expressed: 114 genes were significantly down- and 21 genes were significantly upregulated in COPD (FDR < 0.01)

Nasal epithelial gene expression levels of 135 genes were significantly altered in individuals with COPD versus controls (FDR <0.01; 21 upregulated and 114 downregulated). The top-10 most significantly upregulated genes were *SHROOM1*, *STARD13*, *CMTM1*, *SHC4*, *RRBP1*, *MUC1*, *ARHGEF16*, *TEP1*, *TRIM3* and *GPRC5C*. The top-10 most significantly downregulated genes were *NPHP1*, *CFAP206*, *C11orf70*, *CCDC113*, *CSE1L*, *FAM83B*, *LTV1*, *GMNN*, *SERPINB5* and *AKAP14*. Figure 1 shows a heatmap of all 135 significantly differentially expressed genes between COPD patients and controls, while Table E1 in online Additional file 1 presents all 135 differentially expressed genes.

COPD patients were recruited at multiple study sites within the Netherlands, whereas controls were recruited in the UMCG only. To exclude the possibility that our results were due to study site-specific factors in nasal epithelial sampling, we performed a sub-analysis in nasal epithelial samples derived from individuals with COPD recruited in the UMCG (*n* = 12) and comparing them to the non-COPD controls also recruited from the UMCG (*n* = 22). We also compared individuals with COPD recruited at other study sites (*n* = 19) with the non-COPD controls recruited in the UMCG. We used GSEA to compare COPD-associated gene expression in non-UMCG COPD patients with COPD-associated gene expression in UMCG COPD patients. GSEA showed that the COPD-associated gene expression profiles were comparable between UMCG and non-UMCG COPD patients (FDR_GSEA_ < 0.001; online Additional file 1: Figure E2). In line with the above GSEA results, plots of the principal component analysis including only COPD patients revealed an even distribution of subjects across all study recruitment sites (online Additional file 1: Figure E3), indicating that the study site where the nasal epithelial brushes were collected did not have an effect on gene expression levels.

### Comparison of COPD-associated gene expression in the nasal and bronchial epithelium

We assessed whether COPD-associated gene expression in the nasal epithelium is related to the COPD-associated gene expression changes in the bronchial epithelium. To this end, we explored the direct overlap between COPD-associated genes in nasal and bronchial epithelium in comparator cohort 1. Of the 135 genes significantly differentially expressed in nasal epithelium, 9 out of 21 genes that were significantly upregulated with COPD in nasal epithelium (FDR < 0.01), were also significantly upregulated with COPD in bronchial epithelium (FDR < 0.01). Of the 114 genes significantly downregulated with COPD in nasal epithelium, 19 were also significantly downregulated with COPD in bronchial epithelium (FDR < 0.01; online Additional file 1: Table E2). By chance, we would have expected an overlap of 1 gene at a FDR < 0.01, yet we identified 28 overlapping genes in nasal and bronchial epithelium (Chi square statistic *p*-value <0.01), providing suggestive evidence that our findings are not mere due to chance.

**Fig. 2:**
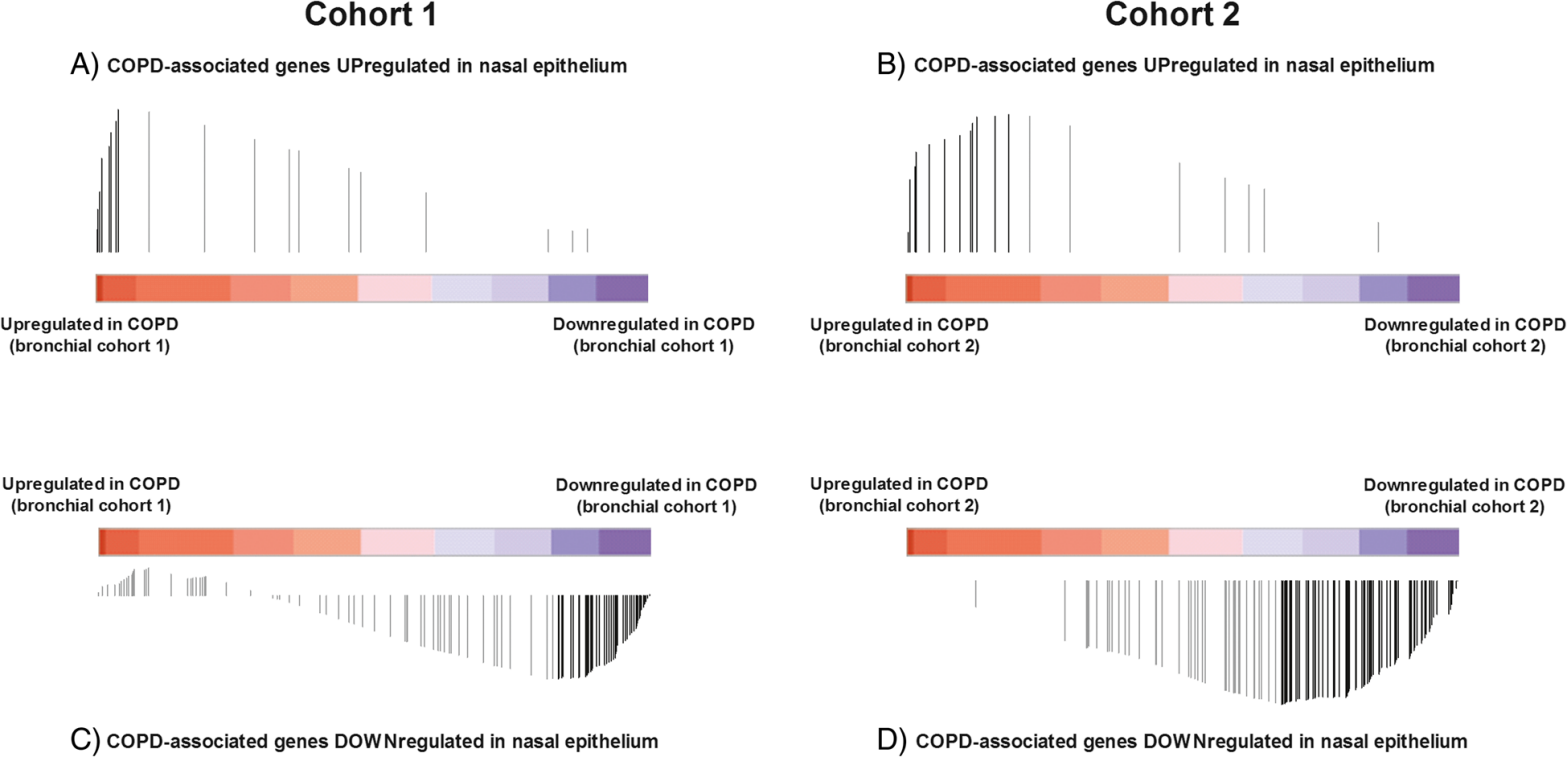
Gene set enrichment analysis showing that nasal gene expression associated with COPD resembles bronchial gene expression. The colored bars represent the ranked t-values of the association of bronchial gene expression with COPD of ~20.000 genes: red represents a positive association whereas blue represents a negative association with COPD. The black vertical lines each represent a significantly differentially expressed gene in nasal epithelium, which are ordered across the ranked bronchial genes. The height of the black lines represents the running enrichment scores of the gene set enrichment analysis. Significant differentially expressed genes at a FDR cut-off of <0.01 are shown. **a** Upregulated genes in nasal epithelium (*n* = 21) were significantly enriched among upregulated genes in bronchial epithelium in cohort 1, **b** Upregulated genes in nasal epithelium (*n* = 21) were significantly enriched among upregulated genes in bronchial epithelium in cohort 2, **c** Downregulated genes in nasal epithelium (*n* = 114) were significantly enriched among downregulated genes in bronchial epithelium in cohort 1, **d** Downregulated genes in nasal epithelium (*n* = 114) were significantly enriched among genes downregulated in bronchial epithelium in cohort 2

Next, we used GSEA to compare COPD-associated nasal epithelial gene expression with COPD-associated bronchial epithelial gene expression in the 2 independent comparator cohorts of subjects with and without COPD. Genes significantly upregulated in nasal epithelium of individuals with COPD were significantly enriched among genes upregulated in bronchial epithelium in COPD in both cohorts (FDR_GSEA_ < 0.001, Fig. 2a and b). Similarly, genes significantly downregulated in nasal epithelium in COPD were significantly enriched among genes downregulated in bronchial epithelium in COPD (FDR_GSEA_ < 0.001, Fig. 2c and d).

### Similarities in pathway enrichment among COPD-associated gene expression in nasal and bronchial epithelium

We identified 6 KEGG pathways that were significantly enriched (FDR_GSEA_ ≤ 0.25) in the nasal epithelium from the identification cohort and in the bronchial epithelium of both comparator cohorts (Table 2). These included 2 pathways enriched among genes upregulated in COPD (O-glycan biosynthesis and glycosphingolipid biosynthesis – lacto and neolacto series) and 4 pathways enriched among genes downregulated in COPD (RNA degradation, DNA replication, propanoate metabolism and tight junction) (online Additional file 1: Tables E3 and E4). Additionally, gene ontology enrichment analyses confirmed significant enrichment of downregulated genes involved in cell cycle and translation, such as negative regulation of G2/M transition of mitotic cell cycle and translational initiation, as well as pathways involved in ciliary function (online Additional file 1: Table E5). For upregulated genes in nasal epithelium, no significant biological processes were identified. These results may reflect a slower rate of epithelium renewal and ciliary dysfunction in COPD.

**Table 2:** Common KEGG pathways and leading-edge genes associated with COPD identified by GSEA (FDR_GSEA_ ≤ 0.25) in nasal and bronchial epithelium

	Nasal		Bronchial (cohort 1)		Bronchial (cohort 2)	
	t-value	*p* value^‡^	t-value	FDR *p* value	t-value	*p* value^‡^
Enriched pathways for genes upregulated in COPD						
O-glycan biosynthesis (KEGG)		0.01^#^		<0.01^#^		0.02^#^
*GALNT6*	3.00	<0.01	3.21	0.02	1.57	0.13
*GALNT12*	3.25	<0.01	3.32	0.01	3.83	<0.01
*B3GNT6*	2.51	0.02	3.55	<0.01	2.92	<0.01
*ST6GALNAC1*	2.65	0.01	3.44	<0.01	1.91	0.07
Glycosphingolipid biosynthesis (KEGG)^a^		0.04^#^		<0.01^#^		0.11^#^
*FUT3*	2.13	0.04	6.76	<0.01	2.68	0.01
*FUT6*	2.72	0.01	6.22	<0.01	2.77	0.01
*B4GALT4*	2.11	0.04	4.15	<0.01	0.12	0.90
*B3GNT3*	3.32	<0.01	5.06	<0.01	1.36	0.19
Enriched pathways for genes downregulated in COPD						
DNA replication (KEGG)		<0.01^#^		0.09^#^		0.19^#^
*RFC3*	−2.36	0.02	−3.82	<0.01	−2.43	0.02
RNA degradation (KEGG)		<0.01^#^		0.06^#^		0.06^#^
Propanoate metabolism (KEGG)		0.13^#^		0.20^#^		0.09^#^
*LDHB*	−3.93	<0.01	−3.88	<0.01	−1.12	0.28
*ACADM*	−2.71	0.01	−6.06	<0.01	−2.14	0.04
*ALDH3A2*	−2.53	0.02	−4.57	<0.01	−1.15	0.26
Tight junction (KEGG)		0.13^#^		0.21^#^		0.24^#^
*CLDN16*	−2.33	0.02	−3.56	<0.01	−2.76	0.01
*CTNNB1*	−3.33	<0.01	−3.18	0.02	−1.65	0.11
*EPB41L2*	−2.64	0.01	−6.03	<0.01	−1.67	0.11

Above leading-edge genes of the 6 significantly enriched KEGG pathways in all three cohorts were significantly differentially expressed between COPD patients and controls both in nasal and bronchial epithelium (cohort 1). The t-value reflects the direction and the strength of the difference in expression of a gene between COPD and controls, *FDR* False discovery rate, ^#^FDR_GSEA_ q value; ^‡^nominal *p*-value. ^a^lacto and neolacto series

We aimed to identify genes that play an important role in these 6 KEGG pathways in nasal and bronchial epithelium. Using GSEA we identified the leading-edge subset of genes (online Additional file 1: Figure E4) for each of the pathways significantly enriched in the three cohorts, and took the overlap of the leading edge genes from these analyses that were also significantly differentially expressed both in nasal epithelium (*p*-value < 0.05) and in bronchial epithelium (cohort 1; FDR < 0.05) (online Additional file 1: Table E6 and E7). Fifteen genes fulfilled above criteria, suggesting they may play a role in COPD (Table 2).

## Discussion

We performed gene expression profiling of the nasal epithelium in smoking individuals with and without COPD and identified a nasal epithelial gene expression profile associated with COPD. Furthermore, we provide suggestive evidence that the COPD-associated nasal gene expression profile overlaps with the COPD-associated bronchial gene expression profile, supporting the concept of a ‘united airway field of injury’. These findings suggest that sampling of nasal epithelium may be useful to study underlying mechanisms of COPD and to develop easily accessible non-invasive biomarkers for disease classification, prognosis and therapeutic monitoring.

There is a high need for an epigenetic biomarker that provides information on disease activity of respiratory diseases, but studies addressing this are limited. A recent study by Obeidat et al. explored expression of 127 emphysema-related genes in lung tissue, bronchial epithelium and peripheral blood in relation to lung function (FEV_1_ and FEV_1_/FVC) [14]. Of the 40 genes associated with lung function in peripheral blood (FDR 0.1), 29 (73%) were overlapping with lung function-associated genes in lung tissue. Of these 29 genes, 4 (13%) had a similar direction in blood and lung tissue. Additionally, they found 13 (33%) lung-function associated genes overlapping between peripheral blood and bronchial epithelium, of which 8 (20%) exhibited changes in similar direction. Of interest, Obeidat et al. used the same bronchial epithelium cohort as investigated in our study [4]. We found that out of 135 genes associated with COPD in nasal epithelium, 28 genes (21%) overlapped with genes associated with COPD in bronchial epithelium in similar direction (FDR 0.01), which is in line with the results of Obeidat et al. Nevertheless there are some considerable differences between the studies. In the first place, our study reports results of a genome wide gene expression analysis to identify COPD-associated genes, while Obeidat et al. conducted a targeted analysis of 127 genes known to be related to emphysema in lung tissue. The targeted approach might have increased the chance of finding differentially expressed genes, but, on the other hand, it disregards expressed genes that are related to features of COPD other than emphysema, such as small airways disease and bronchitis. Additionally, we investigated genes associated with COPD as dichotomous outcome, while Obeidat et al. did not investigate COPD as a disease entity, but investigated FEV_1_ and FEV_1_/FVC as continuous measures. The genes identified by Obeidat et al. are therefore more likely to be related to the severity of airway obstruction while the genes identified in our study are likely to be related to COPD in general. Of importance, we applied a stringent FDR of 0.01 while the former study applied an FDR of 0.1, which might have resulted in more false-positive findings among genes identified in peripheral blood. Also, since smoking status significantly affects gene expression, we only selected current smokers for our gene expression analysis in nasal epithelium, while Obeidat et al. investigated both current- and ex-smokers. In conclusion, both gene expression in nasal epithelium and peripheral blood appear promising as biomarkers in COPD. Future studies should address the direct comparison of gene expression in nasal epithelium and blood in relation to COPD, in which special attention should be given to a similar study design. These studies will be necessary to determine which of these tissues is most suitable to serve as a biomarker in respiratory diseases.

A number of the genes identified in the present study have been previously considered as candidates that associate with features of COPD, such as *MUC1*, *CREB3L1*, *DSP, NPHP1*, *CFAP206* and *CCDC113* [15–28]. Among the top-10 significantly upregulated genes in nasal epithelium of COPD patients is *Mucin 1* (*MUC1*). *MUC1* is a membrane-associated mucin which is expressed in nearly all human glandular epithelial cells including the airway epithelium [15]. It is thought that *MUC1* exerts anti-inflammatory effects upon infection with pathogens by suppressing pro-inflammatory cytokines such as Tumor Necrosis Factor-alpha (TNF-α) [16, 17]. Of interest, plasma and sputum levels of KL-6, a glycoprotein classified as a human *MUC1* mucin, are higher in COPD patients than healthy smokers and non-smokers [18], which is in line with our findings as we found *MUC1* to be upregulated in nasal epithelium of COPD patients. Additionally, cAMP Responsive element Protein-Like 1 (*CREB3L1*) is a transcription factor involved in the unfolded protein response during endoplasmic reticulum stress. It has been proposed that *CREB3L1* has a central role in mucus production, since it is associated with *MUC5AC* expression, an important mucin secreted by the airway epithelium [19]. Furthermore, *CREB3L1* contributes to collagen-containing extracellular matrix production upon simulation with transforming growth factor beta (TGF-β) [20]. Increased mucus production and airway remodeling are key features of COPD [21, 22], therefore upregulation of *CREB3L1* can be envisaged to play a role in the pathophysiology of COPD.

Among the top-10 significantly downregulated genes in nasal epithelium in COPD are nephrocystin 1 (*NPHP1*), cilia and flagella associated protein 206 (*CFAP206*) and coiled-coil domain containing 113 (*CCDC113)*. These 3 genes have in common that they are all involved in cilliagenesis or cilia function. For example, nephrocystin 1 is localized at the ciliary transition zone of respiratory cilia [23]. Next, *CFAP206* has recently been identified as a player in cilium motility through its role in the assembly of the axonemal radial spokes [24]. Finally, *CCDC113* is a centrosome-associated protein and has a function in cilia formation as depletion of *CCDC113* in retinal pigmented epithelial (RPE1) cells led to reduction of cilium formation [25]. In COPD, ciliary function is impaired which leads to decreased mucociliary transport [26]. We found cilia-associated genes to be downregulated in COPD, which might contribute to this process. Another COPD-associated downregulated gene in nasal epithelium is *Desmoplakin (DSP)*, a gene recently discovered to be associated with COPD in a large GWAS [27]. Of interest, in the latter study COPD-linked variants in *DSP* were associated with decreased expression of *DSP* in lung tissue based on expression quantitative trait loci (eQTL) analyses, compatible with our results showing decreased expression in nasal epithelium of COPD patients. *DSP* is one of the important components of desmosomes, a structure that is important in (epithelial) cell adhesion and barrier function [28]. Although studies on the functional role of *DSP* in COPD are lacking, one could speculate that downregulation of *DSP* and, therefore, decreased epithelial barrier function contributes to the mechanisms underlying COPD.

We identified 6 KEGG-pathways that are significantly enriched for genes associated with COPD in both nasal and bronchial epithelium. We identified 4 KEGG pathways that are universally and significantly downregulated in COPD: RNA degradation, DNA replication, propanoate metabolism and tight junction. Of interest, our gene ontology enrichment analysis confirmed downregulation of pathways involved in translation and cell cycle, such as translational initiation and negative regulation of G2/M transition of mitotic cell cycle. It has been demonstrated that cell proliferation is reduced in bronchial epithelial cells and fibroblasts of patients with emphysema compared to controls [29–31], which is in line with our results from the KEGG and gene ontology pathway analyses.

Two pathways are significantly upregulated in both nasal and bronchial epithelium: O-linked glycan biosynthesis and glycosphingolipid biosynthesis. O-linked glycan biosynthesis is a posttranslational process during which carbohydrates (glycans) are attached to proteins. Interestingly, mucin-type O-glycans are the most common form of glycans present in humans [32]. Mucins are highly glycosylated proteins, which form the core structure of mucus. It could be speculated that the observed upregulation of the O-linked biosynthesis pathway in epithelial cells contributes to increase in mucus production, one of the key features of COPD pathophysiology. Glycosphingolipids are membrane lipids (ceramide) to which a glycan is attached. They function as structure elements in the cell membrane and as mediators in cell-cell interaction [33]. Recent studies of our group have shown that concentrations of (glyco)sphingolipids are increased in sputum of COPD patients [34]. Another study describing gene expression in peripheral blood mononuclear cells of COPD patients showed that the sphingolipid metabolism pathway is significantly upregulated in COPD individuals [35]. Additionally, higher blood (glyco)sphingolipids levels are associated with more extensive emphysema and more frequent exacerbations [36]. In combination with our novel findings in nasal epithelial cells, the role of glycosphingolipids appears important in COPD, and the exact contribution to (sub)phenotypes of COPD and targets for possible treatments needs to be further unraveled.

Fifteen genes were of specific interest as they were associated with COPD in both nasal and bronchial epithelium and additionally contributed to significantly enriched pathways associated with COPD. Of the upregulated genes, fucosyltransferase 3 (*FUT3*) and fucosyltransferase 6 (*FUT6*) are involved in the modification of proteins and lipids, i.e. the attachment of fucose. Fucosylation is increasingly recognized as an important player in cell-cell communication [37, 38]. For example, TNF-α, a proinflammatory cytokine, increases expression of *FUT3* and *FUT6* in human bronchial mucosa, suggesting a role for fucosyltransferases in airway inflammation [39]. Of interest, expression of *FUT3* and *FUT6* is associated with the major airway mucin *MUC5AC* [19]. Of the downregulated genes, Replication Factor C-3 (*RFC3*) forms, together with its family members *RFC 1*, *RFC2*, *RFC4* and *RFC5*, a protein complex involved in the regulation of DNA replication and DNA damage repair. *RFC* complexes can function as checkpoints that delay DNA replication in case of DNA damage [40, 41]. Of interest, *RFC1*, *RFC2* and *RFC4* were significantly downregulated (nominal *p* value <0.05) in nasal epithelium as well; however, only *RFC1* was also significantly downregulated in bronchial epithelium. Downregulation of *RFC3* has been associated with lung-, gastric- and colorectal cancer [42, 43], but it might also play a role in COPD as oxidative stress due to cigarette smoke induces DNA damage.

The strength of our study is the use of an identification cohort to determine a COPD-associated nasal gene expression signature and the verification of this signature in two independent cohorts. We selected only current smokers for the analyses, which led to a true reflection of COPD-induced gene expression, hereby avoiding contamination of the results caused by differences in smoking status among patients. Furthermore, by selecting pathways and genes involved in all three cohorts, the probability is high that our findings reflect true biological mechanisms. A limitation of our study is the use of inhaled corticosteroids (ICS) among COPD patients, which was absent in healthy controls. Although ICS use confounds the primary analysis of nasal gene expression in this study, we addressed this issue by assessing whether COPD-associated nasal gene expression changes are similar to that observed in the bronchial airway using an independent dataset of 238 individuals (comparator cohort 1) where only a minority of individuals with COPD used ICS. Another possible limitation of our study is that we compared nasal epithelium of severe COPD patients with bronchial epithelium of moderate-to-severe COPD patients. It would be of added value to study matched nasal and bronchial samples of the same individual, in order to obtain the most appropriate comparison between nasal and bronchial gene expression. These data were not available in our study. However, despite the dissimilarity in COPD severity of the patients with either nasal or bronchial samples, we still found overlap in gene expression between the nose and the bronchus, suggesting a ‘shared’ gene expression signature that is relevant to COPD, regardless of disease severity. It is possible that distinct COPD phenotypes, such as emphysema, small airway disease and chronic bronchitis, have their own specific gene expression changes next to this shared signature, which needs to be explored in future research.

## Conclusion

In conclusion, we demonstrate that the nasal epithelium is a suitable site to detect COPD-associated gene expression alterations. Of interest, we show supportive evidence that this nasal epithelial gene expression profile overlaps with COPD-associated bronchial epithelial gene expression. Our findings underscore the hypothesis that the upper and lower airways have a shared COPD-associated gene expression profile, a so-called ‘united airway field of injury’. Thus nasal gene expression, that is feasible given the ease of access to nasal epithelium, provides the opportunity to explore other applications in future research, such as the diagnosis of distinct molecular phenotypes of COPD and monitoring of disease progression and interventions.

## Additional file

Additional file 1: Supplement details on study design and additional results. (PDF 1068 kb)

## Abbreviations

COPD: Chronic Obstructive Pulmonary Disease
FDR: False Discovery Rate
FEV_1_: Forced Expiratory Volume in 1 s
FVC: Forced Vital Capacity
GSEA: Gene Set Enrichment Analysis
ICS: Inhaled Corticosteroids
KEGG: Kyoto Encyclopedia of Genes and Genomes
PC: Principal Component
RIN: RNA Integrity Number
UMCG: University Medical Center Groningen
