# Supplementary material for: Nasal gene expression differentiates COPD from controls and overlaps bronchial gene expression
Source: Respir Res. 2017 Dec 21;18:213. doi: 10.1186/s12931-017-0696-5 (PMC5740586; doi:10.1186/s12931-017-0696-5)
Supplement: Additional file 1: — Supplement details on study design and additional results. (PDF 1068 kb) [file 12931_2017_696_MOESM1_ESM.pdf]

Online Supplement

Methods

Identification cohort: nasal epithelial brushes

COPD patients: COPD patients participated in a multicenter, randomized, longitudinal study with the aim to compare the efficacy of beclomethasone/formoterol and budesonide/formoterol (FAIR study, ClinicalTrials.gov registration number NCT01351792). Exclusion criteria were the presence of an asthma diagnosis, long-term oxygen therapy, pregnancy, clinically unstable concurrent disease (as judged by the investigator), a COPD exacerbation within 2 months prior to the first study visit and reversibility of the FEV_1 >15% and >200mL of initial FEV_1.

Controls: Controls participated in a cross-sectional study with the aim to obtain normal values of inflammatory variables (NORM study, ClinicalTrials.gov registration number NCT00848406). Normal pulmonary function was defined by a FEV_1/FVC > lower limit of normal, absence of bronchial hyperresponsiveness and reversibility of the FEV_1% predicted to salbutamol <10%. For the present study, we used only data of controls > 40 years. Current smokers had a smoking history of at least 10 pack years. Exclusion criteria were the use of inhaled or oral corticosteroids, upper respiratory tract infection, clinically unstable concurrent disease (as judged by the investigator) or pregnancy.

Comparator cohorts: bronchial epithelial brushes

For bronchial brushes (cohort 1), the same linear regression model as published previously was used to identify differentially expressed genes between COPD and controls[1].

For bronchial brushes (cohort 2), the same linear regression model as used for the nasal gene expression analysis was used to identify differentially expressed genes between COPD and controls.

For both comparator cohorts, bronchial airway gene expression was ranked according to the strength and direction of the association with COPD, and compared to the set of genes significant altered in the nasal epithelial of individuals with COPD compared to those without COPD at FDR < 0.01.

Nasal epithelial brushing

Nasal epithelial brushes in COPD patients were performed at the first study visit with subjects using their daily medication without having received study treatment. First, subjects were asked to blow their nose in order to remove mucus. To numb the nasal mucous membrane, 1 ml lidocaine 1% was sprayed in the right nostril. The lateral area underneath the inferior turbinate was brushed for 3 seconds. Next, the brush was placed in an Eppendorf tube containing RNA-protect fluid (Qiagen, Hilden, Germany) and stored at -80° Celsius until processing.

RNA isolation

Total RNA was isolated from nasal brushes using the miRNeasy kit (Qiagen, Hilden, Germany) according to the protocol of the manufacturer. In short, QIAzol Lysis Reagent was added to the samples to induce lysis. Then, chloroform was added and after centrifuging the sample, the aqueous phase was mixed with 100% ethanol and transferred to a RNeasy® Mini column. The sample was washed once with RWT buffer fluid and two times with RPE buffer fluid. Finally, after adding RNAse-free water, the sample was centrifuged and the RNA fractions

were eluted from the column. Quantity and purity of the RNA was assessed with a NanoDrop 1000 UV-Vis spectrophotometer (Thermo Scientific, Breda, Netherlands) and RNA integrity of the fractions was assessed with an Agilent 2100 BioAnalyzer.

RNA processing and microarray hybridization

To minimize technical variation due to batch effects, we evenly distributed nasal epithelial samples from COPD patients and healthy controls across the different batches, thereby taking into account gender, age and smoking status.

In short, 200ng high molecular weight RNA (i.e. >200 nucleotides) from nasal epithelial brushes was reverse transcribed (Whole Transcript cDNA Sythesis Kit, Affymetrix, Santa Clara, CA). The obtained cDNA was used for *in vitro* transcription (IVT) (Whole Transcript cDNA Amplification Kit, Affymetrix, Santa Clara, CA), purification (GeneChip Sample Cleanup Module, Affymetrix, Santa Clara, CA) and reverse transcription resulting in single-stranded DNA with incorporation of dUTP. Using uracil DNA glycosylase (UDG), and apurinic/apyrimidinic endonuclease 1 (APE 1), the single-stranded DNA was fragmented and labeled with DNA Labeling Reagent which is covalently linked to biotin with terminal deoxynucleotidyl transferase (TdT) (Whole Transcript Terminal Labeling Kit, Affymetrix, Santa Clara, CA). Quality control of IVT and cDNA fragmentation was performed using the mRNA Nano Assay in the Agilent 2100 BioAnalyzer.

Microarray hybridization to Affymetrix Human Gene 1.0 ST Arrays was performed at the Boston University Microarray Core Facility. The labeled cDNA was hybridized to Affymetrix Human Gene 1.0 ST Arrays for 16 to 18 hours in the GeneChip Hybridization Oven 640 at 45°Celsius with rotation at 60 r.p.m. Washing and staining of the hybridized samples was

performed using the Affymetrix Fluidics Station 450 (Hybridization Washing and Staining Kit, Affymetrix, Santa Clara, CA): the samples were first stained with streptavidin (SAPE), followed by administration of a biotinylated goat anti-streptavidin antibody in order to induce signal amplification and finally a second SAPE staining. Immediately after staining, the microarrays were scanned using Affymetrix GeneArray Scanner 3000 7G Plus (Affymetrix, Santa Clara, CA).

Quality control, data normalization and principal component analysis

Normalization was performed with the Robust Multichip Average algorithm using the 'affy' package of 'R' statistical software and Entrez Gene Chip Definition File hugene10sthsentrezgcdf 17.1.0.

The quality of the microarray hybridization was assessed by means of Normalized Unscaled Standard Error (NUSE) plots and Relative Log Expression (RLE) plots as previously described [1]. To account for technical variation within the microarray data after the quality control, a principal component (PC) analysis was performed on the normalized microarray data of the COPD patients and controls (nasal epithelium) and microarray data of cohort 2 (bronchial epithelium) together. To prevent filtering out important clinical variables with the PC analysis, we first adjusted all data for known confounders (age, sex, smoking status, RNA integrity number (RIN)), disease specific variables (FEV_1% predicted) and tissue type (i.e. nasal or bronchial). Next, we performed a PC analysis on the residuals of this analysis to identify PCs accounting for technical variation.

The number of principal components that together explained at least 50% of the variance of the data, were included as covariates in further analyses. This approach resulted in the inclusion of the first 4 principal components, which together explained 52% of the variance.

Results

Identification cohort: nasal epithelial brushes

Nasal samples from 36 current smokers with COPD and 23 current smokers without COPD were hybridized to microarrays. We excluded 1 microarray from a patient with COPD from the analysis due to missing smoking history information, and 5 additional microarrays (4 COPD patients and 1 control) based on quality metrics.

Figure E1

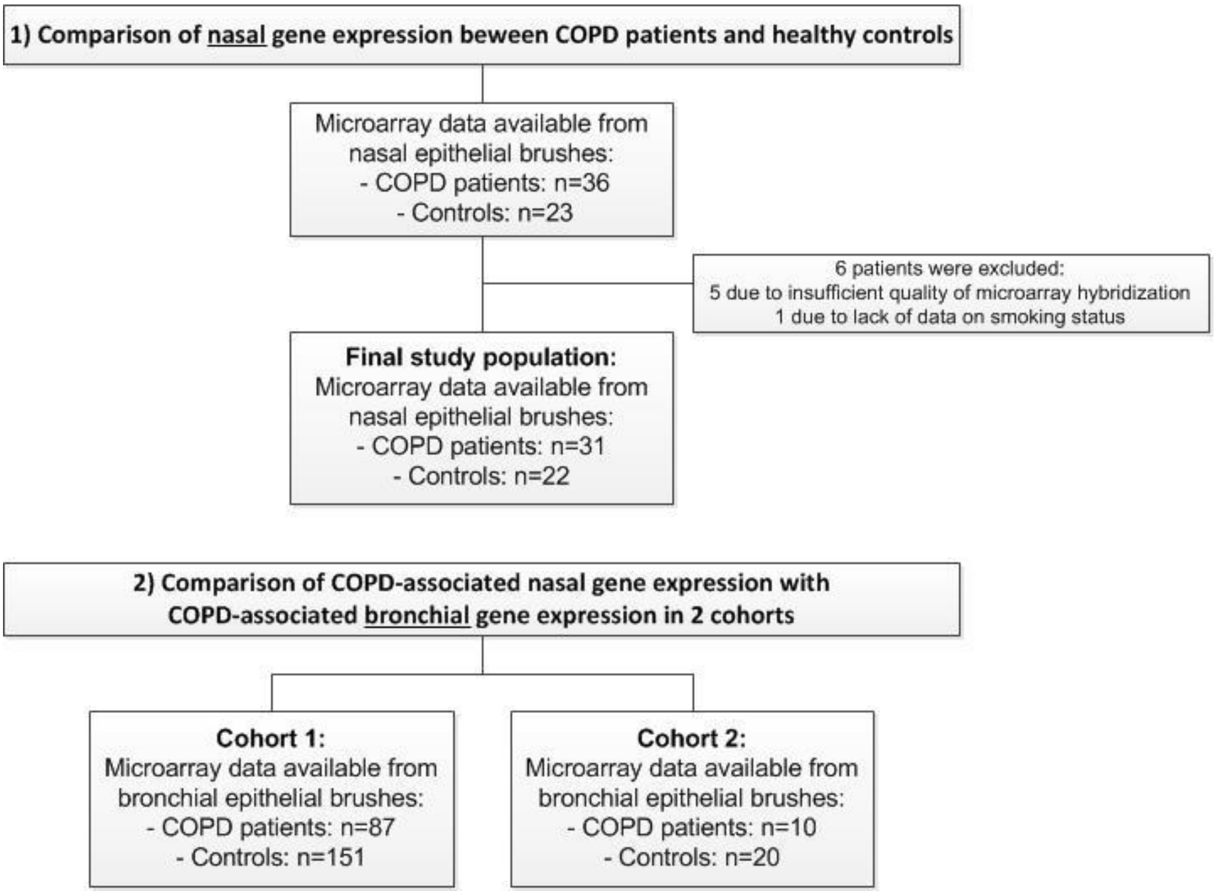
1) Comparison of nasal gene expression beween COPD patients and healthy controls
Microarray data available from nasal epithelial brushes:
- COPD patients: n=36
- Controls: n=23
6 patients were excluded:
5 due to insufficient quality of microarray hybridization
1 due to lack of data on smoking status
Final study population:
Microarray data available from nasal epithelial brushes:
- COPD patients: n=31
- Controls: n=22
2) Comparison of COPD-associated nasal gene expression with COPD-associated bronchial gene expression in 2 cohorts
Cohort 1:
Microarray data available from bronchial epithelial brushes:
- COPD patients: n=87
- Controls: n=151
Cohort 2:
Microarray data available from bronchial epithelial brushes:
- COPD patients: n=10
- Controls: n=20

Table E1: COPD-associated up- and downregulated genes in nasal epithelium (n=135)

Upregulated genes (n=21)

gene name	t value	FDR p value
SHROOM1	5.23	0.0026
STARD13	5.08	0.0035
CMTM1	5.01	0.0040
SHC4	4.92	0.0046
RRBP1	4.88	0.0050
ARHGEF16	4.85	0.0051
MUC1	4.86	0.0051
TEP1	4.77	0.0055
TRIM3	4.75	0.0057
GPRC5C	4.69	0.0062
SDF4	4.66	0.0065
LOC727721	4.58	0.0076
ZNF521	4.53	0.0085
CAPN10	4.54	0.0085
CREB3L1	4.50	0.0088
MYOM1	4.50	0.0089
CHPF2	4.47	0.0091
MAN1B1	4.48	0.0091
LEPRE1	4.46	0.0093
ANKRD10-IT1	4.43	0.0096
DCAF8	4.44	0.0096

Downregulated genes (n=114)

gene name	t value	FDR p value
NPHP1	-7.21	0.0001
CFAP206	-7.17	0.0001
C11orf70	-6.41	0.0006
CCDC113	-6.12	0.0010
CSE1L	-6.10	0.0010
FAM83B	-6.03	0.0011
LTV1	-5.95	0.0011
GMNN	-5.95	0.0011
SERPINB5	-5.72	0.0019
AKAP14	-5.70	0.0019
LRRC49	-5.66	0.0019
CDKN2AIP	-5.66	0.0019
CAPS	-5.54	0.0024
RPAP3	-5.53	0.0024
HSPB1	-5.51	0.0024
TEKT1	-5.48	0.0024
PAK1IP1	-5.48	0.0024
APPL1	-5.43	0.0024
KIFAP3	-5.43	0.0024
CCDC65	-5.42	0.0024
IQCA1	-5.41	0.0024
EIF3M	-5.41	0.0024
CDC20	-5.36	0.0025
RAB38	-5.35	0.0025
RSPH1	-5.35	0.0025
MDH1B	-5.34	0.0025
ADGB	-5.33	0.0025
AK7	-5.31	0.0025
ZNF474	-5.31	0.0025
CAPSL	-5.28	0.0026
WDR66	-5.25	0.0026
CRABP2	-5.25	0.0026
NEK10	-5.24	0.0026
CCDC39	-5.24	0.0026
NR1D2	-5.23	0.0026
CCDC176	-5.22	0.0026
KPNA3	-5.21	0.0026
DUSP11	-5.15	0.0031
WDR96	-5.14	0.0031
DSC2	-5.13	0.0032
KRT13	-5.08	0.0035
C10orf107	-5.07	0.0035
RABL5	-5.06	0.0035
LDLRAD1	-5.06	0.0035
EIF2S1	-5.04	0.0037
LCA5	-5.02	0.0039
YBX3	-5.00	0.0040
MTA3	-4.97	0.0043
GMPS	-4.97	0.0043
APEX1	-4.96	0.0044
SERPINB4	-4.94	0.0045
CETN2	-4.93	0.0045
ENO4	-4.91	0.0048
C15orf26	-4.88	0.0050
MORN2	-4.88	0.0050

ATP5C1	-4.86	0.0051
HIST1H3B	-4.84	0.0052
DSP	-4.83	0.0053
ARMC3	-4.82	0.0055
ERH	-4.81	0.0055
NAE1	-4.79	0.0055
PBK	-4.79	0.0055
GSTA2	-4.79	0.0055
HIST1H2BM	-4.79	0.0055
MYL12B	-4.78	0.0055
HIST1H1B	-4.77	0.0055
CDKL1	-4.77	0.0055
MMP10	-4.77	0.0055
EFCAB1	-4.77	0.0055
DPM1	-4.76	0.0055
S100A2	-4.76	0.0055
HIST1H3G	-4.76	0.0055
SPA17	-4.73	0.0058
PSMG2	-4.73	0.0058
MRPL9	-4.71	0.0062
CDC5L	-4.70	0.0062
UQCRC2	-4.70	0.0062
SULT2B1	-4.69	0.0062
SLC25A5	-4.69	0.0062
APOO	-4.67	0.0065
WDR16	-4.66	0.0065
DSC3	-4.66	0.0065
KRT5	-4.65	0.0066
HN1	-4.64	0.0067
FAM183A	-4.62	0.0072
WDR63	-4.61	0.0072
MTMR2	-4.61	0.0073
ARMC4	-4.60	0.0074
CLCA4	-4.59	0.0074
STX2	-4.58	0.0076
CDK1	-4.56	0.0082
C8orf37	-4.54	0.0085
IQUB	-4.54	0.0085
GTPBP8	-4.53	0.0085
VDAC3	-4.52	0.0086
KRT14	-4.52	0.0087
MNS1	-4.51	0.0087
APOBEC3B	-4.51	0.0087
RPL22L1	-4.51	0.0087
KIAA1430	-4.48	0.0091
LRRIQ1	-4.48	0.0091
PIFO	-4.48	0.0091
C1orf158	-4.46	0.0093
PDE12	-4.46	0.0093
LARP7	-4.45	0.0095
ANXA1	-4.45	0.0095
BNC1	-4.44	0.0095
KCNRG	-4.44	0.0096
MORN5	-4.43	0.0096
PKP1	-4.43	0.0097
SPATA18	-4.42	0.0097
PSMA1	-4.42	0.0098
SERPINB13	-4.42	0.0098
S100A10	-4.41	0.0098

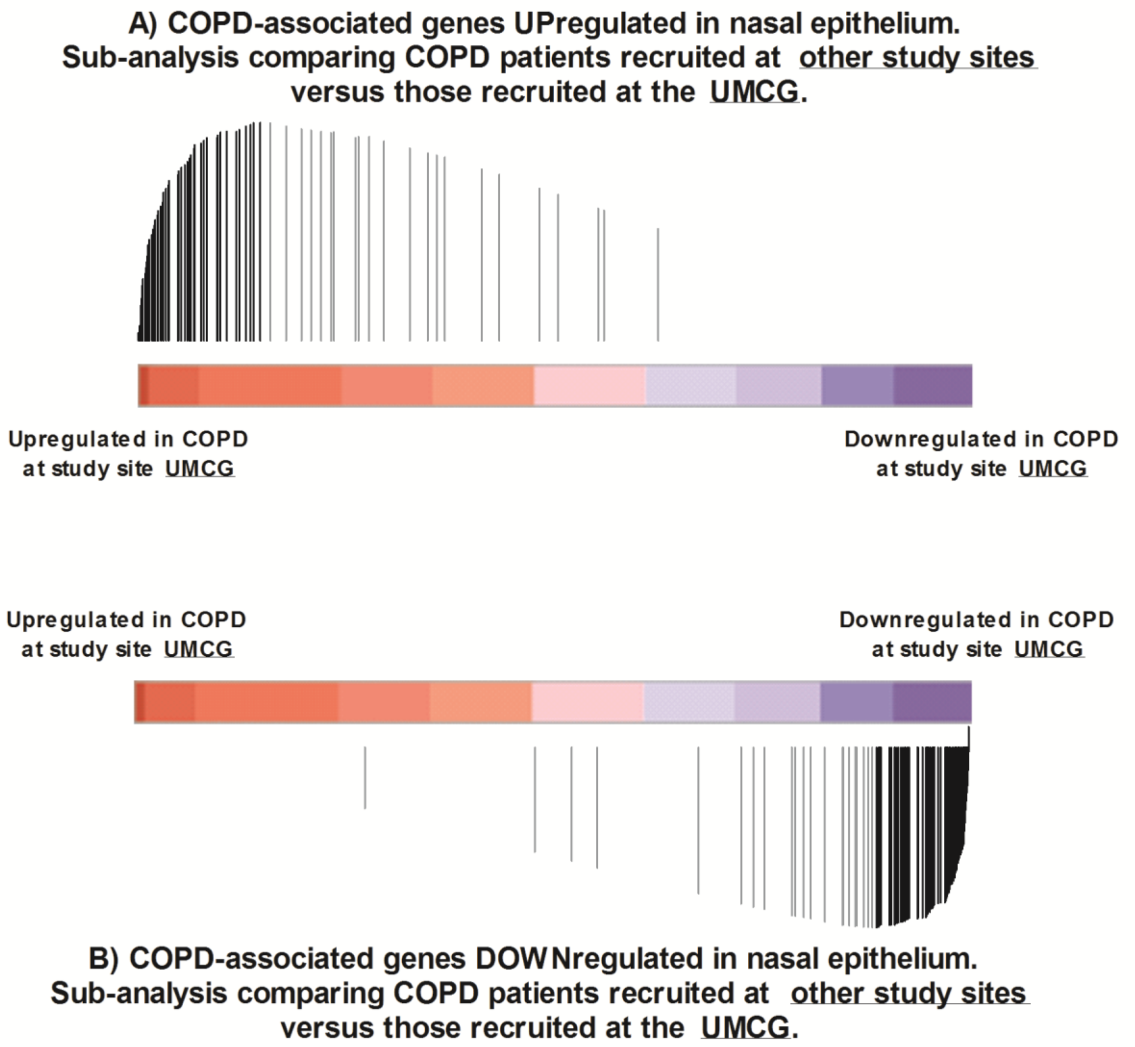

Figure E2: Gene set enrichment analysis showing that COPD-associated nasal gene expression in the subset of COPD patients recruited at other study sites, is comparable with COPD patients recruited at the UMCG. Gene expression of UMCG COPD patients was ranked according to the strength and direction of the association with COPD, and compared to the set of genes significant altered in the nasal epithelial of COPD patients from other study sites at FDR < 0.1. A) Upregulated COPD-associated genes in nasal epithelium in the subset of COPD patients from other study sites are significantly enriched among upregulated genes in the subset of COPD patients from the UMCG (FDR_{GSEA} <0.001); B) Downregulated COPD-associated genes in nasal epithelium in the subset of COPD patients from other study sites are significantly enriched among downregulated genes in the subset of COPD patients from the UMCG (FDR_{GSEA} <0.001).

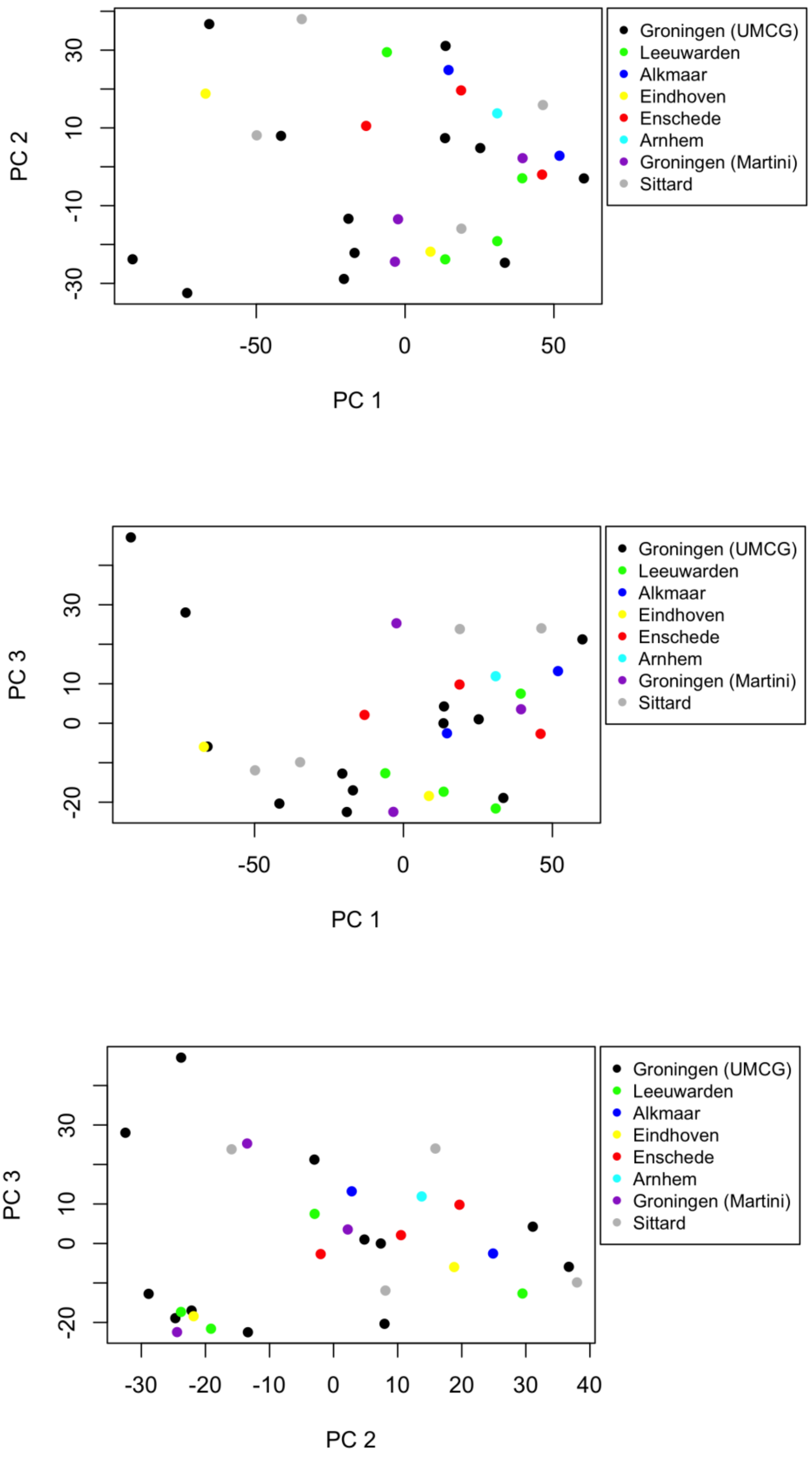

Figure E3: Plots of principal component analysis of only COPD patients colored by study site. PC1= principal component 1; PC2=principal component 2; PC3=principal component 3.

Table E2: Overlapping COPD-associated genes (FDR<0.01) in both nasal and bronchial epithelium (cohort 1)

Upregulated genes (n=9)	Downregulated genes (n=19)
ARHGEF16	*AK7*
CAPN10	*ARMC3*
CHPF2	*ARMC4*
CREB3L1	*C6orf165*
GPRC5C	*C8orf37*
MUC1	*CCDC113*
RRBP1	*CDKL1*
SDF4	*CETN2*
TEP1	*CSE1L*
	ENO4
	GSTA2
	IQCA1
	KCNRG
	KIFAP3
	LRRC49
	MDH1B
	PIFO
	TEKT1
	WDR16

Table E3: KEGG pathways enriched for genes that are UPregulated in COPD (FDR_{GSEA} ≤0.25) in nasal and bronchial epithelium (cohort 1 and cohort 2)

Nasal epithelium	FDR q-val
KEGG_TASTE_TRANSDUCTION	0.00
KEGG_GLYCOSAMINOGLYCAN_DEGRADATION	0.01
**KEGG_O_GLYCAN_BIOSYNTHESIS**	0.01
**KEGG_GLYCOSPHINGOLIPID_BIOSYNTHESIS_LACTO_AND_NEOLACTO_SERIES**	0.04
KEGG_OTHER_GLYCAN_DEGRADATION	0.08
KEGG_ADIPOCYTOKINE_SIGNALING_PATHWAY	0.07
KEGG_GLYCOSAMINOGLYCAN_BIOSYNTHESIS_KERATAN_SULFATE	0.09
KEGG_LYSOSOME	0.11
KEGG_GLYCOSPHINGOLIPID_BIOSYNTHESIS_GANGLIO_SERIES	0.13
KEGG_NITROGEN_METABOLISM	0.13
KEGG_OLFACTORY_TRANSDUCTION	0.18
KEGG_NEUROACTIVE_LIGAND_RECEPTOR_INTERACTION	0.18
KEGG_N_GLYCAN_BIOSYNTHESIS	0.21
KEGG_GLYCOSAMINOGLYCAN_BIOSYNTHESIS_HEPARAN_SULFATE	0.21

Bronchial epithelium (cohort 1)	FDR q-val
**KEGG_O_GLYCAN_BIOSYNTHESIS**	0.00
**KEGG_GLYCOSPHINGOLIPID_BIOSYNTHESIS_LACTO_AND_NEOLACTO_SERIES**	0.00
KEGG_P53_SIGNALING_PATHWAY	0.01
KEGG_APOPTOSIS	0.01
KEGG_GLYCOSAMINOGLYCAN_BIOSYNTHESIS_KERATAN_SULFATE	0.01
KEGG_ENDOCYTOSIS	0.01
KEGG_LINOLEIC_ACID_METABOLISM	0.01
KEGG_N_GLYCAN_BIOSYNTHESIS	0.01
KEGG_GRAFT_VERSUS_HOST_DISEASE	0.02
KEGG_CHRONIC_MYELOID_LEUKEMIA	0.03
KEGG_NATURAL_KILLER_CELL_MEDIATED_CYTOTOXICITY	0.03
KEGG_INOSITOL_PHOSPHATE_METABOLISM	0.03
KEGG_SMALL_CELL_LUNG_CANCER	0.04
KEGG_FC_GAMMA_R_MEDIATED_PHAGOCYTOSIS	0.04
KEGG_NEUROTROPHIN_SIGNALING_PATHWAY	0.05
KEGG_T_CELL_RECEPTOR_SIGNALING_PATHWAY	0.05
KEGG_BLADDER_CANCER	0.05
KEGG_ETHER_LIPID_METABOLISM	0.05
KEGG_PANCREATIC_CANCER	0.05
KEGG_EPITHELIAL_CELL_SIGNALING_IN_HELICOBACTER_PYLORI_INFECTION	0.05
KEGG_TYPE_I_DIABETES_MELLITUS	0.05
KEGG_VIRAL_MYOCARDITIS	0.05
KEGG_CYTOSOLIC_DNA_SENSING_PATHWAY	0.05
KEGG_RETINOL_METABOLISM	0.05
KEGG_PHOSPHATIDYLINOSITOL_SIGNALING_SYSTEM	0.05
KEGG_ARACHIDONIC_ACID_METABOLISM	0.05
KEGG_TOLL_LIKE_RECEPTOR_SIGNALING_PATHWAY	0.06
KEGG_AUTOIMMUNE_THYROID_DISEASE	0.06
KEGG_REGULATION_OF_ACTIN_CYTOSKELETON	0.06
KEGG_VIBRIO_CHOLERAE_INFECTION	0.06
KEGG_GLYOXYLATE_AND_DICARBOXYLATE_METABOLISM	0.07
KEGG_PROSTATE_CANCER	0.07
KEGG_ANTIGEN_PROCESSING_AND_PRESENTATION	0.07
KEGG_INSULIN_SIGNALING_PATHWAY	0.07
KEGG_ACUTE_MYELOID_LEUKEMIA	0.07
KEGG_PATHWAYS_IN_CANCER	0.07
KEGG_NOD_LIKE_RECEPTOR_SIGNALING_PATHWAY	0.07
KEGG_HUNTINGTONS_DISEASE	0.07
KEGG_ALLOGRAFT_REJECTION	0.08
KEGG_AMINO_SUGAR_AND_NUCLEOTIDE_SUGAR_METABOLISM	0.08
KEGG_LEISHMANIA_INFECTION	0.08
KEGG_CHEMOKINE_SIGNALING_PATHWAY	0.08
KEGG_MAPK_SIGNALING_PATHWAY	0.09
KEGG_ERBB_SIGNALING_PATHWAY	0.10
KEGG_PRIMARY_IMMUNODEFICIENCY	0.10
KEGG_METABOLISM_OF_XENOBIOTICS_BY_CYTOCHROME_P450	0.11
KEGG_VEGF_SIGNALING_PATHWAY	0.11
KEGG_ALZHEIMERS_DISEASE	0.12
KEGG_PARKINSONS_DISEASE	0.12
KEGG_CYTOKINE_CYTOKINE_RECEPTOR_INTERACTION	0.11
KEGG_RENAL_CELL_CARCINOMA	0.11
KEGG_AMYOTROPHIC_LATERAL_SCLEROSIS_ALS	0.12
KEGG_NON_SMALL_CELL_LUNG_CANCER	0.13
KEGG_B_CELL_RECEPTOR_SIGNALING_PATHWAY	0.13

Bronchial epithelium (cohort 2)	FDR q-val
KEGG_GLYCOSAMINOGLYCAN_BIOSYNTHESIS_CHONDROITIN_SULFATE	0.01
**KEGG_O_GLYCAN_BIOSYNTHESIS**	0.02
KEGG_NEUROACTIVE_LIGAND_RECEPTOR_INTERACTION	0.03
KEGG_GLYCOSAMINOGLYCAN_BIOSYNTHESIS_HEPARAN_SULFATE	0.09
KEGG_LINOLEIC_ACID_METABOLISM	0.09
**KEGG_GLYCOSPHINGOLIPID_BIOSYNTHESIS_LACTO_AND_NEOLACTO_SERIES**	0.11
KEGG_GLYCOSAMINOGLYCAN_DEGRADATION	0.10
KEGG_GLYCOSPHINGOLIPID_BIOSYNTHESIS_GANGLIO_SERIES	0.13

KEGG_INTESTINAL_IMMUNE_NETWORK_FOR_IGA_PRODUCTION	0.13
KEGG_STARCH_AND_SUCROSE_METABOLISM	0.14
KEGG_AXON_GUIDANCE	0.15
KEGG_ENDOMETRIAL_CANCER	0.16
KEGG_COMPLEMENT_AND_COAGULATION_CASCADES	0.16
KEGG_ONE_CARBON_POOL_BY_FOLATE	0.16
KEGG_GLIOMA	0.16
KEGG_SPHINGOLIPID_METABOLISM	0.17
KEGG_GNRH_SIGNALING_PATHWAY	0.17
KEGG_GLYCEROPHOSPHOLIPID_METABOLISM	0.16
KEGG_GALACTOSE_METABOLISM	0.17
KEGG_VASOPRESSIN_REGULATED_WATER_REABSORPTION	0.17
KEGG_ADIPOCYTOKINE_SIGNALING_PATHWAY	0.17
KEGG_FRUCTOSE_AND_MANNOSE_METABOLISM	0.18
KEGG_PATHOGENIC_ESCHERICHIA_COLI_INFECTION	0.18
KEGG_SYSTEMIC_LUPUS_ERYTHEMATOSUS	0.18
KEGG_BASE_EXCISION_REPAIR	0.19
KEGG_THYROID_CANCER	0.19
KEGG_DORSO_VENTRAL_AXIS_FORMATION	0.19
KEGG_GAP_JUNCTION	0.20
KEGG_JAK_STAT_SIGNALING_PATHWAY	0.21
KEGG_STEROID_HORMONE_BIOSYNTHESIS	0.21
KEGG_PROTEASOME	0.21
KEGG_MELANOMA	0.22
KEGG_GLYCOSAMINOGLYCAN_BIOSYNTHESIS_HEPARAN_SULFATE	0.22
KEGG_NOTCH_SIGNALING_PATHWAY	0.23
KEGG_CELL_CYCLE	0.23
KEGG_PYRIMIDINE_METABOLISM	0.23

Table E4: KEGG pathways enriched for genes that are DOWNregulated in COPD (FDR_{GSEA} ≤0.25) in nasal and bronchial epithelium (cohort 1 and cohort 2)

**Nasal epithelium**	FDR q-val	**Bronchial epithelium (cohort 1)**	FDR q-val	**Bronchial epithelium (cohort 2)**	FDR q-val
KEGG_RIBOSOME	0.00	KEGG_MISMATCH_REPAIR	0.06	KEGG_SYSTEMIC_LUPUS_ERYTHEMATOSUS	0.01
KEGG_CELL_CYCLE	0.00	KEGG_NUCLEOTIDE_EXCISION_REPAIR	0.04	KEGG_ANTIGEN_PROCESSING_AND_PRESENTATION	0.01
KEGG_PROTEASOME	0.00	**KEGG_RNA_DEGRADATION**	0.06	KEGG_DORSO_VENTRAL_AXIS_FORMATION	0.02
KEGG_SYSTEMIC_LUPUS_ERYTHEMATOSUS	0.00	KEGG_VALINE_LEUCINE_AND_ISOLEUCINE_DEGRADATION	0.06	KEGG_GRAFT_VERSUS_HOST_DISEASE	0.06
KEGG_SPLICEOSOME	0.00	**KEGG_DNA_REPLICATION**	0.09	KEGG_VIRAL_MYOCARDITIS	0.06
KEGG_PARKINSONS_DISEASE	0.00	KEGG_OLFACTORY_TRANSDUCTION	0.15	KEGG_AUTOIMMUNE_THYROID_DISEASE	0.05
KEGG_HUNTINGTONS_DISEASE	0.00	KEGG_PRIMARY_BILE_ACID_BIOSYNTHESIS	0.20	KEGG_CELL_CYCLE	0.06
**KEGG_DNA_REPLICATION**	0.00	**KEGG_TIGHT_JUNCTION**	0.21	KEGG_ALLOGRAFT_REJECTION	0.05
KEGG_OXIDATIVE_PHOSPHORYLATION	0.00	**KEGG_PROPANOATE_METABOLISM**	0.20	**KEGG_RNA_DEGRADATION**	0.06
KEGG_CITRATE_CYCLE_TCA_CYCLE	0.00			KEGG_PRION_DISEASES	0.08
**KEGG_RNA_DEGRADATION**	0.00			KEGG_PROTEASOME	0.08
KEGG_NUCLEOTIDE_EXCISION_REPAIR	0.00			KEGG_ENDOMETRIAL_CANCER	0.08
KEGG_ALZHEIMERS_DISEASE	0.00			KEGG_COLORECTAL_CANCER	0.08
KEGG_OOCYTE_MEIOSIS	0.00			KEGG_OOCYTE_MEIOSIS	0.08
KEGG_PATHOGENIC_ESCHERICHIA_COLI_INFECTION	0.00			KEGG_CELL_ADHESION_MOLECULES_CAMS	0.08
KEGG_P53_SIGNALING_PATHWAY	0.00			KEGG_INTESTINAL_IMMUNE_NETWORK_FOR_IGA_PRODUCTION	0.08
KEGG_MISMATCH_REPAIR	0.00			KEGG_PATHOGENIC_ESCHERICHIA_COLI_INFECTION	0.07
KEGG_GLUTATHIONE_METABOLISM	0.00			KEGG_THYROID_CANCER	0.08
KEGG_SMALL_CELL_LUNG_CANCER	0.02			KEGG_LEISHMANIA_INFECTION	0.07
KEGG_PROGESTERONE_MEDIATED_OOCYTE_MATURATION	0.03			KEGG_ADHERENS_JUNCTION	0.07
KEGG_PYRUVATE_METABOLISM	0.03			KEGG_TYPE_I_DIABETES_MELLITUS	0.07
KEGG_BASAL_TRANSCRIPTION_FACTORS	0.04			KEGG_RNA_POLYMERASE	0.07
KEGG_BASE_EXCISION_REPAIR	0.04			KEGG_GLYCOSYLPHOSPHATIDYLINOSITOL_GPI_ANCHOR_BIOSYNTHESIS	0.08
KEGG_AMINOACYL_TRNA_BIOSYNTHESIS	0.06			KEGG_GLUTATHIONE_METABOLISM	0.08
KEGG_BUTANOATE_METABOLISM	0.09			KEGG_RENAL_CELL_CARCINOMA	0.08
KEGG_METABOLISM_OF_XENOBIOTICS_BY_CYTOCHROME_P450	0.10			**KEGG_PROPANOATE_METABOLISM**	0.09
KEGG_COLORECTAL_CANCER	0.10			KEGG_ASTHMA	0.09
KEGG_DRUG_METABOLISM_CYTOCHROME_P450	0.10			KEGG_TGF_BETA_SIGNALING_PATHWAY	0.10
KEGG_VALINE_LEUCINE_AND_ISOLEUCINE_DEGRADATION	0.11			KEGG_UBIQUITIN_MEDIATED_PROTEOLYSIS	0.13
KEGG_PYRIMIDINE_METABOLISM	0.12			KEGG_P53_SIGNALING_PATHWAY	0.14
KEGG_GLYCOLYSIS_GLUCONEOGENESIS	0.12			KEGG_VASOPRESSIN_REGULATED_WATER_REABSORPTION	0.15
KEGG_UBIQUITIN_MEDIATED_PROTEOLYSIS	0.13			KEGG_SPHINGOLIPID_METABOLISM	0.15
**KEGG_TIGHT_JUNCTION**	0.13			KEGG_ACUTE_MYELOID_LEUKEMIA	0.15
KEGG_BLADDER_CANCER	0.13			KEGG_BASAL_TRANSCRIPTION_FACTORS	0.15
**KEGG_PROPANOATE_METABOLISM**	0.13			KEGG_T_CELL_RECEPTOR_SIGNALING_PATHWAY	0.16
KEGG_ARRHYTHMOGENIC_RIGHT_VENTRICULAR_CARDIOMYOPATHY_ARVC	0.14			KEGG_NEUROTROPHIN_SIGNALING_PATHWAY	0.16
KEGG_REGULATION_OF_ACTIN_CYTOSKELETON	0.14			KEGG_ALZHEIMERS_DISEASE	0.16
KEGG_GLIOMA	0.15			KEGG_RENIN_ANGIOTENSIN_SYSTEM	0.17
KEGG_ADHERENS_JUNCTION	0.15			KEGG_PEROXISOME	0.17
KEGG_ASTHMA	0.15			KEGG_LEUKOCYTE_TRANSENDOTHELIAL_MIGRATION	0.17
KEGG_PROSTATE_CANCER	0.22			**KEGG_DNA_REPLICATION**	0.19
				KEGG_HUNTINGTONS_DISEASE	0.18
				KEGG_B_CELL_RECEPTOR_SIGNALING_PATHWAY	0.23
				KEGG_HEDGEHOG_SIGNALING_PATHWAY	0.23
				KEGG_ARRHYTHMOGENIC_RIGHT_VENTRICULAR_CARDIOMYOPATHY_ARVC	0.24
				KEGG_MELANOGENESIS	0.24
				**KEGG_TIGHT_JUNCTION**	0.24
				KEGG_PATHWAYS_IN_CANCER	0.25

Table E5: GO term analyses (GOrilla) for genes DOWNregulated in COPD in nasal epithelium (FDR<0.05)

Gene Ontology Process	P-value	FDR q-value
microtubule-based process	1.5E-16	2.24E-12
**rRNA processing**	1.09E-14	8.1E-11
**translational initiation**	2.33E-14	1.15E-10
rRNA metabolic process	1.34E-13	4.98E-10
posttranscriptional regulation of gene expression	3.26E-12	9.69E-9
organelle organization	9.06E-12	2.24E-8
**cilium movement**	1.85E-11	3.93E-8
chromosome segregation	2.47E-11	4.6E-8
mitotic cell cycle process	3.22E-11	5.32E-8
cellular component organization or biogenesis	4.17E-11	6.2E-8
cellular component assembly	4.87E-11	6.59E-8
cellular component organization	1.13E-10	1.4E-7
regulation of cell cycle G2/M phase transition	1.56E-10	1.78E-7
microtubule-based movement	2.76E-10	2.93E-7
cellular macromolecular complex assembly	5.34E-10	5.29E-7
regulation of ubiquitin-protein ligase activity involved in mitotic cell cycle	5.77E-10	5.36E-7
ncRNA processing	7.13E-10	6.24E-7
regulation of mitotic cell cycle phase transition	9.77E-10	8.07E-7
**positive regulation of ubiquitin-protein ligase activity involved in regulation of mitotic cell cycle transition**	1.32E-9	1.03E-6
organelle assembly	1.33E-9	9.92E-7
regulation of cell cycle phase transition	1.53E-9	1.08E-6
regulation of G2/M transition of mitotic cell cycle	1.83E-9	1.24E-6
cell cycle process	3.32E-9	2.15E-6
**negative regulation of ubiquitin-protein ligase activity involved in mitotic cell cycle**	4.64E-9	2.87E-6
positive regulation of ubiquitin protein ligase activity	4.93E-9	2.93E-6
regulation of cell cycle process	8.87E-9	5.07E-6
negative regulation of cell cycle process	1.18E-8	6.52E-6
negative regulation of mitotic cell cycle phase transition	1.4E-8	7.42E-6
negative regulation of ubiquitin protein ligase activity	1.45E-8	7.42E-6
negative regulation of mitotic cell cycle	1.51E-8	7.47E-6
positive regulation of protein ubiquitination involved in ubiquitin-dependent protein catabolic process	1.61E-8	7.72E-6
regulation of ubiquitin protein ligase activity	1.89E-8	8.77E-6
**anaphase-promoting complex-dependent catabolic process**	2.06E-8	9.29E-6
regulation of protein ubiquitination involved in ubiquitin-dependent protein catabolic process	2.12E-8	9.29E-6
regulation of cell cycle	2.76E-8	1.17E-5
cellular macromolecule catabolic process	2.84E-8	1.17E-5
negative regulation of cell cycle phase transition	3.26E-8	1.31E-5
cilium organization	3.57E-8	1.4E-5
regulation of translation	3.77E-8	1.44E-5
negative regulation of ubiquitin-protein transferase activity	4.04E-8	1.5E-5
negative regulation of cell cycle	4.39E-8	1.59E-5
**motile cilium assembly**	4.66E-8	1.65E-5
**chromosome organization**	6.26E-8	2.16E-5
regulation of mitotic cell cycle	6.62E-8	2.24E-5
positive regulation of ubiquitin-protein transferase activity	7.2E-8	2.38E-5
nuclear-transcribed mRNA catabolic process	9.61E-8	3.11E-5
RNA catabolic process	1.11E-7	3.5E-5
negative regulation of macromolecule metabolic process	1.23E-7	3.8E-5
regulation of cellular amide metabolic process	2.45E-7	7.44E-5
mRNA catabolic process	2.83E-7	8.43E-5
ncRNA metabolic process	3.03E-7	8.84E-5
negative regulation of protein modification by small protein conjugation or removal	3.14E-7	8.99E-5
protein-DNA complex assembly	3.36E-7	9.43E-5
**negative regulation of G2/M transition of mitotic cell cycle**	4.09E-7	1.13E-4
single-organism organelle organization	4.35E-7	1.18E-4
mitotic cell cycle	4.72E-7	1.25E-4
negative regulation of cell cycle G2/M phase transition	5.43E-7	1.42E-4
RNA processing	5.74E-7	1.47E-4
viral process	6.92E-7	1.74E-4

Table E5 continued

Gene Ontology Process	P-value	FDR q-value
symbiosis, encompassing mutualism through parasitism	6.92E-7	1.71E-4
establishment of protein localization to endoplasmic reticulum	7.15E-7	1.74E-4
negative regulation of gene expression	7.65E-7	1.84E-4
regulation of ubiquitin-protein transferase activity	7.71E-7	1.82E-4
negative regulation of protein ubiquitination	7.94E-7	1.85E-4
cell division	8.5E-7	1.94E-4
**SRP-dependent cotranslational protein targeting to membrane**	9.02E-7	2.03E-4
positive regulation of cellular protein catabolic process	9.67E-7	2.15E-4
macromolecular complex subunit organization	1.01E-6	2.21E-4
cell projection organization	1.11E-6	2.39E-4
**regulation of mRNA stability**	1.19E-6	2.52E-4
plasma membrane bounded cell projection organization	1.81E-6	3.8E-4
cotranslational protein targeting to membrane	1.84E-6	3.79E-4
protein modification by small protein removal	1.89E-6	3.85E-4
macromolecular complex assembly	1.94E-6	3.9E-4
macromolecule catabolic process	2.3E-6	4.56E-4
interspecies interaction between organisms	2.53E-6	4.95E-4
protein localization to endoplasmic reticulum	2.54E-6	4.9E-4
protein targeting to ER	2.74E-6	5.22E-4
regulation of RNA stability	2.78E-6	5.22E-4
protein-DNA complex subunit organization	2.92E-6	5.43E-4
cell cycle	3.86E-6	7.08E-4
positive regulation of proteolysis involved in cellular protein catabolic process	4.44E-6	8.05E-4
regulation of cellular amine metabolic process	4.72E-6	8.46E-4
**nuclear-transcribed mRNA catabolic process, nonsense-mediated decay**	5.02E-6	8.88E-4
microtubule organizing center organization	5.04E-6	8.81E-4
**translation**	5.63E-6	9.74E-4
plasma membrane bounded cell projection assembly	6.12E-6	1.05E-3
microtubule bundle formation	6.55E-6	1.11E-3
regulation of cellular protein catabolic process	7.03E-6	1.17E-3
**regulation of transcription from RNA polymerase II promoter in response to hypoxia**	7.06E-6	1.17E-3
cell projection assembly	8.21E-6	1.34E-3
negative regulation of transferase activity	8.41E-6	1.36E-3
negative regulation of metabolic process	8.95E-6	1.43E-3
nucleobase-containing compound catabolic process	9.61E-6	1.52E-3

NB: GO terms in bold represent biological processes at the bottom of the hierarchical diagram created by GOrilla, i.e. the most specific pathways. Only pathways with a p-value< p-value < 1E-5 are shown.

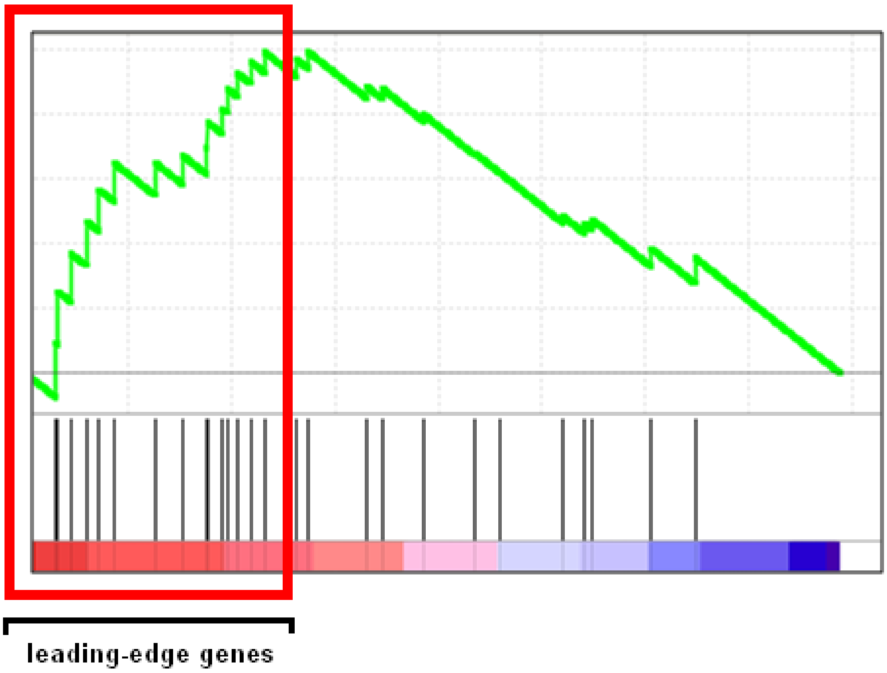

Figure E4: Example of the leading-edge subset, consisting of those genes that contribute the most to the enrichment of pathways; in the figure the leading-edge genes are ranked before the point at which the running sum (represented by the green line) reaches its highest point.

Table E6: Leading-edge genes of the 2 significantly enriched UPregulated KEGG pathways in nasal and bronchial epithelium (cohort 1 and cohort 2)

O-Glycan biosynthesis

Nasal	Bronchial (cohort 1)	Bronchial (cohort 2)
GALNT4	***GALNT4***	***GALNT4***
GALNT5	***GALNT5***	***GALNT5***
GALNT6	***GALNT6***	***GALNT6***
GALNT7	***GALNT7***	***GALNT7***
GALNT12	***GALNT12***	***GALNT12***
B3GNT6	***B3GNT6***	***B3GNT6***
ST6GALNAC1	***ST6GALNAC1***	***ST6GALNAC1***
GCNT3	*GCNT3*	-
GALNT3	-	*GALNT3*
GALNTL6	-	*GALNTL6*
GALNT9	-	*GALNT9*
ST3GAL1	-	*ST3GAL1*
WBSCR17	-	*WBSCR17*
GALNT8	-	-
GALNT10	-	-
GALNT11	-	-
C1GALT1C1	-	-
ST3GAL2	-	-
C1GALT1	-	-
-	*B4GALT5*	-
-	*GALNT14*	-
-	*GCNT1*	-
-	*GALNT2*	-
-	*GALNT1*	-

Glycosphingolipid biosynthesis

Nasal	Bronchial (cohort 1)	Bronchial (cohort 2)
FUT3	***FUT3***	***FUT3***
FUT6	***FUT6***	***FUT6***
ST3GAL3	***ST3GAL3***	***ST3GAL3***
B4GALT4	***B4GALT4***	***ST3GAL4***
B3GNT3	***B3GNT3***	***B3GNT3***
ABO	***ABO***	***ABO***
FUT2	*FUT2*	-
ST3GAL4	*ST3GAL4*	-
B4GALT1	*B4GALT1*	-
B4GALT3	*B4GALT3*	-
FUT4	-	*FUT4*
B3GNT4	-	*B3GNT4*
-	*B4GALT2*	*B4GALT2*
-	*FUT1*	*FUT1*
B3GALT5	-	-
-	*B3GALT1*	-
-	*B3GNT2*	-
-	-	-
-	-	-
-	-	*ST8SIA1*
-	-	*FUT5*

Table E7: Leading-edge genes of the 3 significantly enriched DOWNregulated KEGG pathways in nasal and bronchial epithelium (cohort 1 and cohort 2)

DNA replication

Nasal	Bronchial (cohort 1)	Bronchial (cohort 2)
**POLD2**	**POLD2**	**POLD2**
**PRIM1**	**PRIM1**	**PRIM1**
**RFC3**	**RFC3**	**RFC3**
**RPA1**	**RPA1**	**RPA1**
MCM2	MCM2	-
RFC5	RFC5	-
RFC1	RFC1	-
-	RPA2	RPA2
POLE3	-	POLE3
RFC2	-	RFC2
RFC4	-	RFC4
RNASEH2A	-	RNASEH2A
RNASEH2B	-	RNASEH2B
SSBP1	-	SSBP1
MCM4	-	MCM4
DNA2	-	-
FEN1	-	-
LIG1	-	-
MCM5	-	-
MCM6	-	-
MCM7	-	-
PCNA	-	-
POLA2	-	-
POLD4	-	-
POLE2	-	-
-	PRIM2	-
-	RNASEH1	-
-	RPA3	-
-	-	MCM3
-	-	POLA1

-

Propanoate metabolism

Nasal	Bronchial (cohort 1)	Bronchial (cohort 2)
**SUCLG2**	**SUCLG2**	**SUCLG2**
**HADHA**	**HADHA**	**HADHA**
**LDHB**	**LDHB**	**LDHB**
**ALDH3A2**	**ALDH3A2**	**ALDH3A2**
**ACADM**	**ACADM**	**ACADM**
-	ALDH9A1	ALDH9A1
-	ACAT2	ACAT2
-	MUT	MUT
-	ALDH2	ALDH2
-	ACSS1	ACSS1
-	EHHADH	EHHADH
LDHA	-	LDHA
PCCB	-	PCCB
SUCLG1	-	SUCLG1
SUCLA2	-	-
ACAT1	-	-
-	LDHAL6A	-
-	LOC283398	-
-	MCEE	-
-	ALDH6A1	-
-	-	ABAT
-	-	HIBCH
-	-	LDHC

RNA degradation

Nasal	Bronchial (cohort 1)	Bronchial (cohort 2)
**PAPOLG**	**PAPOLG**	**PAPOLG**
**LSM3**	**LSM3**	**LSM3**
**CNOT1**	**CNOT1**	**CNOT1**
**SKIV2L2**	**SKIV2L2**	**SKIV2L2**
**CNOT10**	**CNOT10**	**CNOT10**
**DCP1B**	**DCP1B**	**DCP1B**
**CNOT6L**	**CNOT6L**	**CNOT6L**
**DDX6**	**DDX6**	**DDX6**
-	WDR61	WDR61
-	ENO2	ENO2
TTC37	TTC37	-
CNOT2	CNOT2	-
DIS3	DIS3	-
PAPOLA	PAPOLA	-
LSM6	LSM6	-
XRN2	XRN2	-
EXOSC9	EXOSC9	-
EXOSC1	EXOSC1	-
EXOSC8	EXOSC8	-
EXOSC2	EXOSC2	-
EXOSC3	-	EXOSC3
EXOSC4	-	EXOSC4
EXOSC5	-	EXOSC5
LSM4	-	LSM4
LSM5	-	LSM5
CNOT8	-	CNOT8
CNOT7	-	CNOT7
HSPD1	-	HSPD1
EXOSC7	-	-
HSPA9	-	-
ENO1	-	-
EDC3	-	-
LSM1	CNOT4	-
-	RQCD1	-
-	PNPT1	-
-	PAPOLB	-
-	DCP2	-
-	ZCCHC7	-
-	EXOSC10	-
-	-	PATL1

Tight junction

Nasal	Bronchial (cohort 1)	Bronchial (cohort 2)
**CLDN16**	**CLDN16**	**CLDN16**
**CTNNB1**	**CTNNB1**	**CTNNB1**
**EPB41L2**	**EPB41L2**	**EPB41L2**
**PPP2CB**	**PPP2CB**	**PPP2CB**
**PRKCZ**	**PRKCZ**	**PRKCZ**
**VAPA**	**VAPA**	**VAPA**
MPP5	MPP5	-
PRKCA	PRKCA	-
MPDZ	MPDZ	-
EPB41L3	EPB41L3	-
OCLN	OCLN	-
PARD3	PARD3	-
-	CLDN8	CLDN8
-	CLDN18	CLDN18
-	JAM3	JAM3
-	PARD6B	PARD6B
-	ZAK	ZAK
-	CLDN9	CLDN9
ASH1L	-	ASH1L
ACTG1	-	ACTG1
ACTN4	-	ACTN4
AKT3	-	AKT3
AMOTL1	-	AMOTL1
CDK4	-	CDK4
CLDN1	-	CLDN1
CLDN4	-	CLDN4
CSNK2B	-	CSNK2B
F11R	-	F11R
MYL9	-	MYL9
PPP2CA	-	PPP2CA
CASK	-	-
MLLT4	-	-
TJP1	-	-
YES1	-	-
GNAI3	-	-
MYH14	-	-
MYH9	-	-
MYL12A	-	-
MYL12B	-	-
NRAS	-	-
PARD6G	-	-
PPP2R2A	-	-

Table E7 continued

Tight junction

Nasal	Bronchial (cohort 1)	Bronchial (cohort 2)
PPP2R2C	-	-
PTEN	-	-
RAB13	-	-
SRC	-	-
-	*CLDN22*	-
-	*PRKCB*	-
-	*MYH10*	-
-	*PRKCD*	-
-	*EXOC4*	-
-	*CSNK2A1*	-
-	*MAGI1*	-
-	*PPP2R2D*	-
-	*PRKCH*	-
-	*PRKCE*	-
-	*CGN*	-
-	*RAB3B*	-
-	*CSNK2A2*	-
-	*MAGI2*	-
-	*INADL*	-
-	-	*MYH2*
-	-	*CLDN3*
-	-	*AKT1*
-	-	*ACTB*
-	-	*MYH11*
-	-	*MYH6*
-	-	*MYH7B*
-	-	*PPP2R1A*
-	-	*SPTAN1*
-	-	*RHOA*

References

1. Steiling K, van den Berge M, Hijazi K, Florido R, Campbell J, Liu G, et al. A dynamic bronchial airway gene expression signature of chronic obstructive pulmonary disease and lung function impairment. Am J Respir Crit Care Med. 2013;187: 933-942.
